# Effect of an intravenous acetaminophen/ibuprofen fixed-dose combination on postoperative opioid consumption and pain after video-assisted thoracic surgery: a double-blind randomized controlled trial

**DOI:** 10.1007/s00464-024-10821-y

**Published:** 2024-04-12

**Authors:** Ho-Jin Lee, Seungeun Choi, Soohyuk Yoon, Susie Yoon, Jae-Hyon Bahk

**Affiliations:** 1 Department of Anesthesiology and Pain Medicine, Seoul National University Hospital, 101 Daehak-ro, Jongno-gu, Seoul, 03080 Republic of Korea; 2 Department of Anesthesiology and Pain Medicine, Seoul National University College of Medicine, 101 Daehak-ro, Jongno-gu, Seoul, 03080 Republic of Korea

**Keywords:** Analgesia, Non-narcotic, Analgesics, Opioid, Perioperative care, Postoperative pain, Thoracic surgery, Video-assisted

## Abstract

**Background:**

Video-assisted thoracoscopic surgery (VATS) often induces significant postoperative pain, potentially leading to chronic pain and decreased quality of life. This study aimed to evaluate the acetaminophen/ibuprofen combination effectiveness in reducing analgesic requirements and pain intensity in patients undergoing VATS.

**Study design:**

This is a double-blinded randomized controlled trial.

**Methods:**

Adult patients scheduled for elective VATS for lung resection were randomized to receive either intravenous acetaminophen and ibuprofen (intervention group) or 100 mL normal saline (control group). Treatments were administered post-anesthesia induction and every 6 h for three cycles. The primary outcome was total analgesic consumption at 24 h postoperatively. Secondary outcomes were cumulative analgesic consumption at 2 and 48 h; analgesic-related side effects at 2, 24, and 48 h; quality of recovery at 24 h and 48 h postoperatively; pain intensity at rest and during coughing; and rescue analgesics use. Chronic postsurgical pain (CPSP) was assessed through telephone interviews 3 months postoperatively.

**Results:**

The study included 96 participants. The intervention group showed significantly lower analgesic consumption at 24 h and 48 h postoperatively (24 h: median difference: − 100 µg equivalent intravenous fentanyl [95% confidence interval (CI) − 200 to − 5 μg], *P* = 0.037; 48 h: median difference: − 140 μg [95% CI − 320 to − 20 μg], *P* = 0.035). Compared to the controls, the intervention group exhibited a significantly lower quality of recovery 24 h post-surgery, with no significant difference at 48 h. All pain scores except for coughing at 48 h post-surgery were significantly lower in the intervention group compared to the controls. No significant differences were observed between the groups in postoperative nausea and vomiting occurrence, hospital stay length, and CPSP.

**Conclusion:**

Perioperative administration of acetaminophen/ibuprofen significantly decreased analgesic needs in patients undergoing VATS, providing an effective postoperative pain management strategy, and potentially minimizing the need for stronger analgesics.

**Supplementary Information:**

The online version of this article (10.1007/s00464-024-10821-y) contains supplementary material, which is available to authorized users.

Video-assisted thoracoscopic surgery (VATS) is widely performed as the standard treatment for early-stage lung cancer, owing to its advantages over thoracotomy. These advantages include less postoperative pain, shorter hospital stay, and a quicker return to daily activities [1, 2]. However, VATS can still induce significant postoperative pain, which arises from damage to the intercostal nerves and thoracic wall muscles due to trocar insertion, as well as intercostal nerve compression or irritation caused by chest tube placement and pleural bleeding [3, 4]. Uncontrolled acute postoperative pain can increase postoperative complications and length of hospital stay, lead to chronic postoperative pain, and result in a long-term decline in quality of life after surgery [5]. Therefore, acute postoperative pain control in patients undergoing VATS is of utmost importance.

Although opioids remain commonly utilized for postoperative pain management, their adverse effects can negatively impact clinical outcomes [6]. These side effects can impede postoperative recovery and increase medical costs [7]. Consequently, multimodal opioid-sparing analgesia has been established as the standard for perioperative pain management for thoracic surgery [8, 9]. In line with this objective, the use of a combination of non-opioid analgesics, each with different mechanisms of action, is recommended [10]. This strategy effectively reduces postoperative pain while concurrently minimizing the side effects associated with opioids.

Among various non-opioid analgesics, the combination of acetaminophen and non-steroidal anti-inflammatory drugs (NSAID) is likely the most widely used and recommended for postoperative pain management for thoracic surgery [9]. They act through mechanisms distinct from opioid analgesics, effectively controlling postoperative pain and reducing postoperative opioid requirement, thereby minimizing opioid-related side effects [11]. A recent intravenous (IV) formulation of 1000 mg acetaminophen and 300 mg ibuprofen (Maxigesic® IV, Kyungbo Pharm, Asan, Korea), released in South Korea, has shown superior pain control in surgical patients compared to either drug alone [12, 13]. However, to our best knowledge, the routine use of a combination of acetaminophen and NSAID for patients undergoing VATS is not yet a common practice in South Korea. Therefore, we aimed to investigate the effects of perioperative IV administration of a combination of 1000 mg acetaminophen and 300 mg ibuprofen on postoperative opioid requirements and pain intensity in patients undergoing VATS for lung cancer resection. This study is expected to provide crucial information regarding the efficacy of administering a combination of acetaminophen and NSAIDs in patients undergoing VATS. This potentially aids the broader application of this analgesic combination in patients undergoing surgery.

## Materials and methods

### Study design and participants

This prospective randomized controlled trial (RCT) was approved by the relevant Institutional Review Board and registered at ClinicalTrials.gov (NCT05366777, registration date May 4, 2022). The study design and reporting of its findings adhered to the consolidated standards of reporting Trials (CONSORT) guidelines. All participants provided written informed consent prior to enrollment.

Adult patients aged 19–80 years scheduled for elective unilateral VATS for segmentectomy or lobectomy between October 2022 and July 2023 were included in the study. The exclusion criteria were as follows: (1) American Society of Anesthesiologists (ASA) physical status III or above; (2) chronic pain lasting over 3 months; (3) current pregnancy or lactation; (4) allergies to study drugs; (5) medical or psychological conditions potentially affecting treatment response; (6) inability to comprehend the study protocol or provide informed consent; and (7) planned postoperative analgesia not included in the study protocol. Participants were excluded from the study if they withdrew consent, surgery was canceled, unexpected thoracotomy was needed, or mechanical ventilation exceeded 2 h post-surgery. The participants were asked to complete the Korean version of the Quality of Recovery-15 (QoR-15K) questionnaire.

The baseline characteristics of the participants were recorded, including age, sex, height, weight, body mass index, ASA physical status, Eastern Cooperative Oncology Group (ECOG) performance status, preoperative QoR-15K score, serum creatinine, liver function tests, type of operation, ports used for VATS, duration of operation, and intraoperative remifentanil consumption.

### Randomization and blinding

After enrollment, the patients were randomly allocated to either the intervention group (acetaminophen/ibuprofen) or the control group. This was performed using block randomization (blocks 4 and 6) in a 1:1 ratio using R software (version 3.5.1, R Foundation for Statistical Computing, Vienna, Austria). The randomization process was overseen by an anesthesiologist who was blinded to the study. The randomization list was forwarded to a nurse who was not involved in the study. This nurse is responsible for preparing and dispensing medications according to the list. The acetaminophen/ibuprofen fixed-dose combination and placebo (normal saline) were indistinguishable in appearance to ensure the blinding of care providers, investigators, patients, and outcome assessors.

### Study intervention

The intervention group received an intravenous infusion of a fixed-dose combination of acetaminophen (1000 mg) and ibuprofen (300 mg), while the control group received a matching volume (100 mL) of normal saline. This was administered over 15 min following induction of general anesthesia and repeated postoperatively every 6 h for three doses.

### Perioperative management

Consistent monitoring and management protocols were applied to all patients, in alignment with the standard care practices of our institution. Prior to surgery, the patient did not receive any premedication. Patients received total intravenous anesthesia with a target-controlled infusion of propofol, remifentanil, and rocuronium for intraoperative neuromuscular blockade. To prevent postoperative nausea and vomiting (PONV), a combination of 0.075 mg palonosetron and 5 mg dexamethasone was administered intravenously during anesthesia induction. Subsequently, each participant received 100 mL of the assigned solution infused for 15 min. Before making the surgical incision, the attending surgeons performed intercostal nerve blockade with 0.25% bupivacaine.

VATS was performed using either a 2-port or 3-port technique. This approach involved making incisions less than 5 cm in length with rib sparing. Lung protective ventilation strategies were applied by adjusting the tidal volumes to 6–8 mL/kg of ideal body weight for two-lung ventilation and 4–6 mL/kg for one-lung ventilation, along with a positive end-expiratory pressure of 5–10 cm H_2_O. Upon completion of the surgery and skin closure, each patient received an intravenous dose of 50 µg fentanyl. Subsequently, an intravenous patient-controlled analgesia device (IV-PCA; Accumate® 1200, Woo Young Meditech, Seoul, Korea) was connected to the patient’s IV line, dispensing fentanyl at a concentration of 20 µg/mL, with a 1 mL bolus dose and a 10-min lock-out interval. Neuromuscular blockade was reversed using 2–4 mg/kg sugammadex, after which the patients were extubated and transferred to the post-anesthesia care unit (PACU).

Postoperative care involved administering 100 mL of the previously allocated solution to each patient every 6 h in three doses. The use of IV-PCA was permitted for those experiencing pain levels of 3 or higher on the numeric rating scale (NRS). In cases where the NRS pain score reached 7 or more, 50 µg of IV fentanyl was provided as the primary rescue analgesia. For patients experiencing PONV, an alternative rescue analgesic, ketorolac tromethamine (30 mg), was administered. Postoperative protocols allowed the resumption of water intake and ward ambulation 6 h after surgery. From this point on, patients were offered either an oral arginine 185 mg/ibuprofen 200 mg tablet or a 5 mg Oxycodone tablet as an additional rescue analgesic. Rescue antiemetics were provided based on patient requests or in response to moderate-to-severe PONV symptoms, with 10 mg metoclopramide administered in the PACU and an initial dose of 0.3 mg ramosetron followed by metoclopramide (10 mg) in the ward if needed. The decision to administer rescue analgesics and antiemetics in both the PACU and ward was made by attending physicians who were blinded to the patient group allocations.

### Outcome measures

The primary outcome measure was the quantity of analgesics consumed within the first 24 h after surgery. The secondary outcome measures were the cumulative analgesic use at 2 and 48 h postoperatively; the intensity of pain both at rest and during coughing, quantified using an 11-point NRS; the frequency of rescue analgesic utilization; the incidence of side effects linked to analgesics, such as nausea, vomiting, the need for rescue antiemetics, sweating, palpitations, and sedation, noted at 2, 24, and 48 h after surgery; and assessments of the QoR-15K at 24 and 48 h postoperatively. The total dose of rescue analgesics was converted to an equivalent intravenous fentanyl dose, based on previous studies [14, 15]. Additionally, patients were monitored for major postoperative complications, as defined by the Clavien–Dindo classification (grade III or higher), throughout the hospital stay. Furthermore, 3 months after surgery, we conducted telephone interviews to assess (1) pain intensity at the surgical site (using the 11-point NRS); (2) the temporal pattern of the pain experienced; (3) any neuropathic components of the pain such as burning, tingling, sharp shocks, hyperalgesia, allodynia, or numbness; and (4) the use of analgesics. All outcomes were evaluated by clinicians who were blinded to the group assignments.

### Statistical analysis

The required sample size for this study was predetermined using G*Power software (version 3.1.9; Düsseldorf, Germany). Drawing on data from a prior study [16], the average fentanyl consumption post-VATS in the initial 24 h was established at 615 (± 293) μg. Anticipating a clinically significant 30% reduction in total fentanyl consumption within the first 24 h for the acetaminophen/ibuprofen group (effect size: 0.630), we determined that 43 patients in each group would provide an 80% probability of detecting a statistical difference using the Mann–Whitney *U* test, with a two-sided *α* level set at 0.05. We aimed to accommodate a potential dropout rate of 10 total for 48 patients per group.

The analysis was conducted according to intention-to-treat principles. The distribution normality of continuous variables was assessed using the Shapiro–Wilk test. These data were presented either as mean (with standard deviation) or median (with interquartile range), and comparisons between groups were made using either the independent *t*-test or Mann–Whitney *U* test. Categorical data, described as frequencies or percentages, were compared using either the *χ*^2^ test or Fisher’s exact test depending on the expected counts. We also calculated effect sizes and 95% confidence intervals. For multiple comparisons, excluding those involving postoperative fentanyl consumption, Bonferroni correction was applied. All statistical analyses were carried out using R software (version 3.6), with all tests being two-sided and a significance threshold of *P* < 0.05.

## Results

**Fig. 1:**
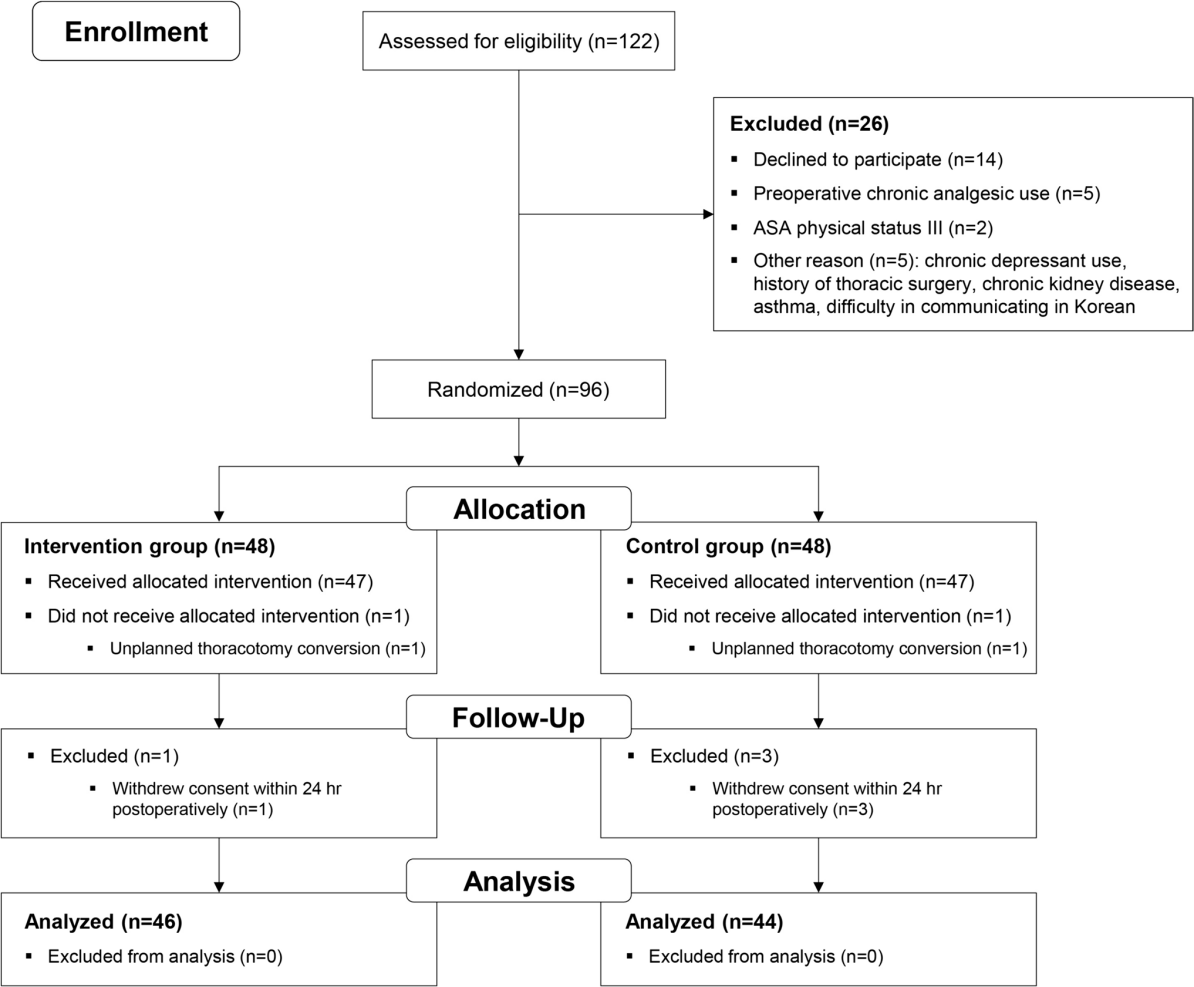
Consolidated Standards of Reporting Trials flow diagram for patient enrollment. *ASA* American Society of Anesthesiologists

Of the 122 patients assessed for eligibility, 96 met the inclusion criteria and were subsequently enrolled and randomly allocated to either the intervention or the control group, as depicted in Fig. 1 between October 2022 and July 2023. Following recruitment, 2 patients from the intervention group and 4 from the control group were excluded on the day of surgery. Consequently, 90 patients were included in the final analysis. The baseline characteristics of the patients are shown in Table 1. There were no significant differences in the characteristics between the two groups.

**Table 1:** Baseline patient characteristics

	Intervention group (*n* = 46)	Control group (*n* = 44)
Female	22 (47.8)	21 (47.7)
Age (years)	63.9 ± 8.3	65.8 ± 7.2
Height (cm)	163.6 ± 7.6	162.6 ± 7.5
Weight (kg)	64.1 ± 10.5	63.0 ± 8.2
BMI (kg/m^2^)	23.9 ± 2.7	23.7 ± 2.1
ECOG performance status, 0/1	45 (97.8)/1 (2.2)	41 (93.2)/3 (6.8)
ASA physical status, I/II	5 (10.9)/41 (89.1)	6 (13.6)/38 (86.4)
Preoperative QoR-15K (0–150)	148 (142 to 150)	149 (144 to 150)
Preoperative creatinine (mg/dL)	0.8 (0.7 to 0.9 [0.6 to 1.5])	0.8 (0.7 to 0.9 [0.6 to 1.5])
Preoperative aspartate transaminase (U/L)	20.0 (17.0 to 24.0 [14.0 to 37.0])	21.5 (18.0 to 25.5 [14.0 to 48.0])
Preoperative alanine transferase (U/L)	16.5 (13.0 to 23.0 [9.0 to 56.0])	18.0 (16.0 to 25.0 [10.0 to 61.0])
Type of operation		
VATS lobectomy	27 (58.7)	33 (75.0)
VATS segmentectomy	19 (41.3)	11 (25.0)
Two-port/three port VATS technique	24 (52.2)/22 (47.8)	19 (43.2)/25 (56.8)
Operation time (min)	95 (80 to 115)	95 (78 to 113)
Intraoperative remifentanil (μg)	836 (704 to 1139)	807 (648 to 996)

The data are expressed as either the mean ± standard deviation, median (interquartile range [range]), or as a count (percentage)

*ASA* American Society of Anesthesiologists, *BMI* body mass index, *ECOG* Eastern Cooperative Oncology Group, *QoR-15K* Korean version of the Quality of Recover-15, *VATS* video-assisted thoracic surgery

**Fig. 2:**
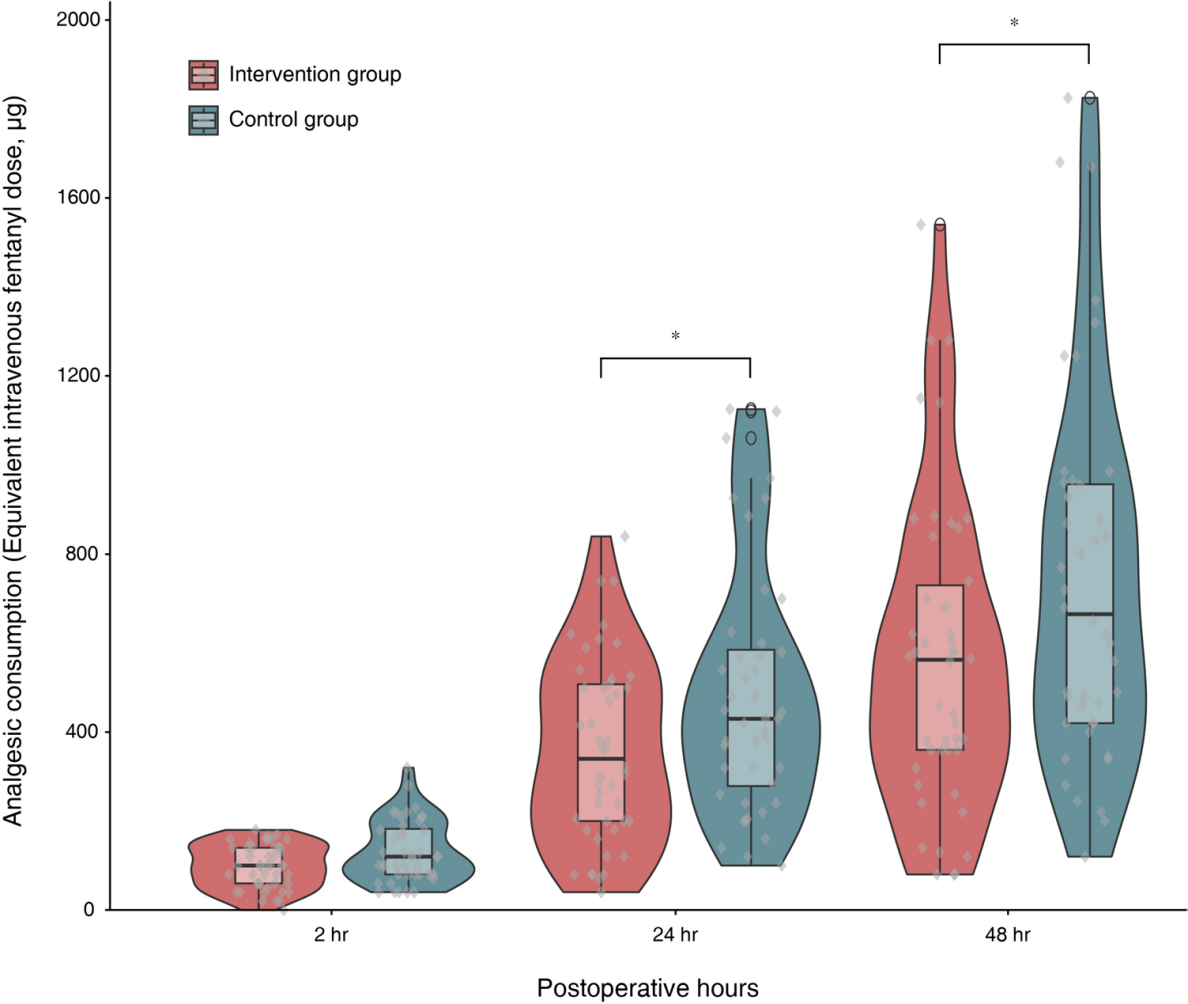
Between-group comparisons of the total intravenous analgesic consumption during the first 2, 24, and 48 h postoperatively. Median and interquartile range of each parameter are presented with boxplot and entire distributions of the measurements are shown with rotated kernel density plot in the intervention (*n* = 46) and control (*n* = 44) groups. Upper and lower whiskers are maximum and minimum values excluding outliers, respectively. Round symbols show the outliers. Scatter plot (diamond symbols) shows the individual data points. *P < 0.05 by Mann–Whitney *U* test

**Fig. 3:**
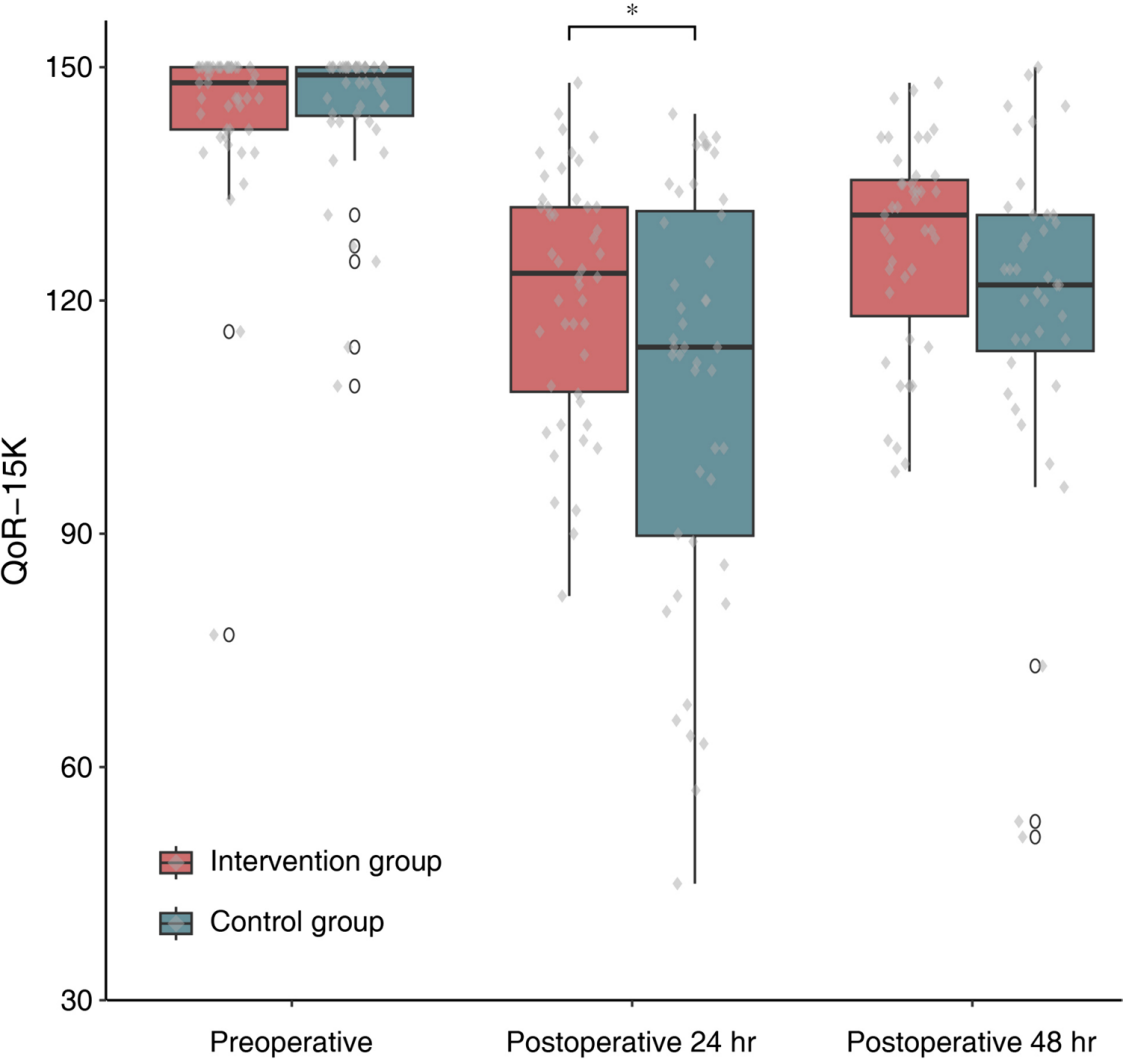
Between-group comparisons of the Korean version of the Quality of Recovery-15 (QoR-15K) score during 24 and 48 h postoperatively. Median and interquartile range of each parameter are presented with boxplot in the intervention (*n* = 46) and control (*n* = 44) groups. Upper and lower whiskers are maximum and minimum values excluding outliers, respectively. Round symbols show the outliers. Scatter plot (diamond symbols) shows the individual data points. *P < 0.05 by Mann–Whitney *U* test. ^a^This included 43 patients in the intervention group and 39 patients in the control group

Figure 2 shows a comparison of the overall analgesic consumption between the two groups. The intervention group showed significantly lower overall analgesic consumption during the first postoperative 24 h (median difference: − 100 μg [95% CI − 200 to − 5 μg], *P* = 0.037) and 48 h (median difference: − 140 μg [95% CI − 320 to − 20 μg], *P* = 0.035) postoperatively. In the intervention group, the quality of recovery assessed by the QoR-15K at 24 h post-surgery was significantly higher than that in the control group (median difference: 9 [95% CI 0 to 19], *P* = 0.046), but no significant difference was observed at 48 h (median difference: 6 [95% CI 0 to 13], *P* = 0.053) as shown in Fig. 3. Table 2 presents a comparison of postoperative outcomes other than intravenous analgesic consumption and quality of recovery. In the intervention group, all pain assessments except for pain during coughing at 48 h post-surgery showed significantly lower scores than those in the control group. The occurrence rates of PONV, length of postoperative hospital stay, postoperative complications, and chronic pain assessed 3 months postoperatively were not significantly different between the two groups.

**Table 2:** Between-group comparison of pain score and other postoperative outcomes

	Intervention group (*n* = 46)	Control group (*n* = 44)	Median difference or relative risk (95% CI)	*P*-value
Pain score at rest, NRS (0–10)				
2 h	2 (2 to 3)	3 (3 to 4)	− 1 (− 1 to 0)	< 0.001
24 h	2 (2 to 3)	3 (3 to 4)	− 1 (− 1 to 0)	< 0.001
48 h	2 (1 to 3)	3 (2 to 3)	− 1 (− 1 to 0)	0.005
Pain score during coughing, NRS (0–10)				
24 h	4 (3 to 5)	5 (4 to 6)	− 1 (− 2 to − 1)	< 0.001
48 h	3 (3 to 4)	4 (3 to 5)	0 (− 1 to 0)	0.070
Rescue analgesic use during postoperative hospital stay				
0–2 h	2 (4.3)	5 (11.4)	0.38 (0.08 to 1.87)	0.235
2–24 h	11 (23.9)	14 (31.8)	0.75 (0.38 to 1.47)	0.406
24–48 h	6 (13.1)	5 (11.4)	1.15 (0.38 to 3.49)	0.808
Rescue fentanyl equivalent dose during postoperative hospital stay (µg)	0 (0 to 0)	0 (0 to 25)	0 (0 to 0)	0.078
Postoperative nausea and vomiting				
0–2 h	2 (4.3)	1 (2.3)	1.91 (0.18 to 20.35)	0.591
2–24 h	10 (21.7)	12 (27.3)	0.80 (0.38 to 1.65)	0.543
24–48 h	7 (15.2)	7 (15.9)	0.96 (0.37 to 2.50)	0.928
Postoperative creatinine (mg/dL)	0.8 (0.7 to 0.9 [6 to 1.3])	0.8 (0.7 to 0.9 [0.6 to 1.2])	–	
Postoperative aspartate transaminase (U/L)	23.0 (21.0 to 26.0 [14.0 to 37.0])	24.0 (19.5 to 27.0 [13.0 to 49.0])	–	
Postoperative alanine transferase (U/L)	16.5 (13.0 to 21.0 [8.0 to 44.0])	16.0 (14.0 to 22.0 [10.0 to 44.0])	–	
Length of postoperative hospital stay (days)	3 (2 to 5)	3 (2 to 4)	0 (− 1 to 1)	0.677
Clavien–Dindo classification, grade III or higher	5 (10.9)	3 (6.8)	1.59 (0.40 to 6.28)	0.504
Chronic postoperative pain at 3 months^a^	14 (37.8)	13 (36.1)	1.05 (0.58 to 1.90)	0.879
Neuropathic pain	2 (5.4)	3 (8.3)	0.65 (0.12 to 3.66)	0.623
NRS 3 or higher	3 (8.1)	3 (8.3)	0.97 (0.21 to 4.51)	0.972

The values are presented as the median (interquartile range [range]) or number (%)

*CI* confidence interval, *NRS* numeric rating scale

^a^This included 37 patients in the intervention group and 36 patients in the control group

## Discussion

In this study, perioperative intravenous administration of a combined acetaminophen/ibuprofen formulation significantly reduced postoperative opioid consumption and pain intensity after VATS for lung cancer resection. This might lead to a significant improvement in the quality of recovery at 24 h postoperatively without serious adverse effects. These results underscore the benefits of incorporating a combination of acetaminophen and ibuprofen into opioid-based pain management in patients undergoing VATS and support the routine use of a combination of acetaminophen and NSAID [8].

While recognizing the importance of comparing the combination drug's effects with its individual components, this study focused on comparisons with placebo, given the already established superior efficacy of the combination drug over each drug alone in surgical patients [17]. A previous meta-analysis has highlighted the enhanced analgesic effects of acetaminophen/NSAID combinations compared to each drug alone, thus advocating for combination therapy in postoperative pain control [18]. Network meta-analysis has also demonstrated the greater effectiveness of these combinations in reducing postoperative opioid use compared to single agents [19]. Although one RCT involving patients undergoing total hip arthroplasty did not find a significant reduction in opioid consumption over 24 h postoperatively with the combined oral administration of both drugs compared to ibuprofen alone, this study also reported that the combination of these two drugs significantly reduced postoperative pain compared to each drug administered individually [20]. Furthermore, a previous study evaluating the combination drug used in our RCT corroborates its superior efficacy in postoperative pain management compared to each component individually [12]. Given these findings and our limited research resources, we purposefully excluded a single-drug group, focusing instead on the comparative effects of the combination drug against opioid-based pain management, to emphasize the utility of these non-opioid analgesics.

While the combination of acetaminophen and NSAIDs has become the standard treatment for postoperative pain management [9], the combination of these two non-opioid analgesics appears to be less widely applied than expected. Although there are no surveys on the use of non-opioid analgesics in surgical patients in South Korea, studies conducted in other countries have shown that, despite strong evidence supporting their use, the application rate of combined acetaminophen/NSAIDs in surgical patients remains low. A study utilizing large nationwide data in the United States found that the average predicted probability of patients undergoing major surgery being prescribed two or more non-opioid analgesics was 54.2%, with tremendous variation in non-opioid analgesic prescriptions among hospitals [21]. A recent study in Ontario, Canada, targeting elderly patients aged 66 and over, found that 21% of all patients were prescribed only opioids within one week after surgery [22]. Although the prescription of non-opioid analgesics showed an increasing trend during the study period, the number of patients receiving a combination of two or more non-opioid analgesics remained low [22]. In addition, according to a survey on perioperative management in thoracic surgery in the UK and Ireland, postoperative use of acetaminophen was reported in 98% of cases, while the use of NSAIDs was only 55% [23]. In a study conducted at a single institution in the United States, similar results were observed where the prescription rate of acetaminophen in surgical patients was over 90%, but the prescription rate of NSAIDs did not exceed 40% [24]. Against this backdrop, we aimed to establish the evidence for perioperative administration of the combined acetaminophen/ibuprofen formulation, with the intention of applying it in clinical practice.

In our study, the combined acetaminophen/ibuprofen formulation not only reduced the need for opioids during the first 24 h postoperatively, but also significantly decreased pain intensity up to 48 h postoperatively. Additionally, these effects might lead to a significant improvement in the quality of postoperative recovery at 24 h postoperatively, with the difference exceeding the clinically meaningful minimal difference previously established for QoR-15K [25]. However, in our study, the observed opioid-sparing effect of the combined acetaminophen/ibuprofen formulation was less pronounced than that previously reported for acetaminophen/NSAID combinations [18, 19]. The perioperative management protocol we applied might have influenced these results. In both groups, propofol-based total intravenous anesthesia was administered, which may have reduced the postoperative opioid requirements [26], thereby diminishing the apparent magnitude of the opioid-sparing effect of the acetaminophen/ibuprofen combination. Additionally, the administration of dexamethasone and the implementation of preemptive intercostal nerve blockade in both groups likely influenced this outcome [27, 28]. Moreover, as no patients in the control group experienced severe postoperative pain under multimodal analgesia, it would have been difficult to add a combined acetaminophen/ibuprofen formulation to influence the incidence of chronic postoperative pain. Furthermore, the combined acetaminophen/ibuprofen formulation did not significantly affect the PONV incidence. This outcome could also be attributed to the use of propofol-based total intravenous anesthesia, dexamethasone, and palonosetron in both groups and the diminished opioid-sparing effect due to the reasons mentioned above, which likely made it challenging for this combination drug to significantly impact the occurrence of PONV in this relatively small study.

However, despite the smaller-than-expected opioid-sparing effect of the combined acetaminophen/ibuprofen formulation, its administration improved the quality of recovery 24 h postoperatively. This improvement was likely due to pain relief rather than an opioid-sparing effect. In our previous study in patients undergoing VATS, the aspect with the most potential for improvement among the QoR-15K items was the one related to postoperative pain, and postoperative pain intensity showed a significant correlation with the quality of recovery as assessed by the QoR-15K [29]. Therefore, it is likely that the reduction in pain due to the additional administration of the combined acetaminophen/ibuprofen formulation contributed significantly to enhancing the overall quality of postoperative recovery, rather than its opioid-sparing effect.

Our study had the following limitations. First, it was a single-center RCT. As previously mentioned, differences in perioperative protocols that can affect postoperative opioid requirements and opioid-related side effects may have influenced the study results. Second, to assess the 24-h efficacy of the acetaminophen/ibuprofen combination compared to the placebo, we started administering the oral acetaminophen/tramadol combination 24 h postoperatively. However, in our institution's protocol, oral analgesics are routinely administered starting 6 h after surgery once fasting is discontinued. Nevertheless, based on our clinical experience, PONV, which is common in patients undergoing VATS, can hinder the early administration of oral analgesics and potentially affect study results. Therefore, we planned to start oral analgesics 24 h postoperatively, following the completion of acetaminophen/ibuprofen combination administration. Finally, although no serious side effects related to the combined acetaminophen–ibuprofen formulation were observed in our study, we excluded patients with conditions susceptible to the side effects of these drugs based on stringent exclusion criteria. Given the rare incidence of side effects associated with these medications, the relatively small number of patients included in this study may not have sufficiently demonstrated the safety of these drugs in surgical patients. Therefore, further studies are required to evaluate the safety profile of this drug.

In conclusion, perioperative IV administration of a combined acetaminophen/ibuprofen formulation demonstrated a notable opioid-sparing and analgesic effect within the framework of multimodal analgesia for patients with lung cancer undergoing VATS. Consequently, this combination is a viable component of multimodal analgesia in these patients. However, our study did not conclusively establish the safety profile of this medication, indicating the need for additional research to assess its safety thoroughly.

### Supplementary Information

Below is the link to the electronic supplementary material.

(PDF 225 kb)
